# Multiple Myeloma and Bisphosphonate-Related Osteonecrosis of the Mandible Associated with Dental Implants

**DOI:** 10.1155/2011/568246

**Published:** 2011-07-02

**Authors:** Luis Junquera, Lorena Gallego, Alejandro Pelaz

**Affiliations:** ^1^Oral and Maxillofacial Surgery, Dental School, University of Oviedo, Catedrático José Serrano Street, 33009 Oviedo, Spain; ^2^Department of Oral and Maxillofacial Surgery, Central University Hospital, 33006 Oviedo, Spain; ^3^Department of Oral and Maxillofacial Surgery, Cabueñes Hospital, 33394 Gijón, Spain

## Abstract

Multiple myeloma (MM) is a malignant plasma cell disorder and more than 30% of patients with this pathology develop osteolytic lesions in the jaw. Either pamidronate or zoledronic acid is recommended in patients with MM who have one or more lytic lesions. However, bisphosphonate-related osteonecrosis of the jaws (BRONJ) has been described as a complication associated with their use. Otherwise, the use of endosseous implants in oral rehabilitation is a well-established procedure, with good long-term success although systemic factors may affect the bone healing around dental implants. We report the first case reported of MM adjacent to a mandibular dental implant in a patient who developed BRONJ in the same area after intravenous zoledronate treatment. We discuss possible pathogeny of this particular and interesting phenomena.

## 1. Introduction

Multiple myeloma (MM) is a malignant plasma cell disorder that accounts for approximately 10% of all hematologic cancers [1]. It develops mainly in men aged from 50 to 80 years, with a mean of 66 years [2]. More than 30% of patients with MM develop osteolytic lesions in the jaw [3]. Those lesions are more frequent in the posterior region of the mandible [4]. The oral manifestations of MM are first signs of the disease in about 14% of the patients and may include swelling, pain, bleeding, mobile teeth, amyloid deposits, root resorption and mobility, labial anesthesia, and jaw radiolucencies and fractures [2, 3]. Histological analysis of bone lesions usually reveals plasmacytoid cells, with round, eccentric nuclei with fine granular chromatin and evident nucleolus, characteristics of a solid malignant hematopoietic neoplasm [1]. 

Restorative treatment of missing teeth via dental titanium implants has a considerable effect on oral health, and quality of life is improved as compared to conventional removable denture prostheses [5]. Various factors have been implicated in the success of implants, but these studies have been largely limited to patients with “normal” hard and soft tissues. Moreover, systemic factors may affect the bone healing around dental implants [6, 7].

Either pamidronate (90 mg intravenously over at least 2 hours every 4 weeks) or zoledronic acid (4 mg intravenously over 15–30 minutes every 4 weeks) are recommended in patients with MM who have one or more lytic lesions on skeletal roentgenograms. The administration of bisphosphonates significantly reduces the number of skeletal events (pathologic fracture, need for irradiation or surgery on bone, and spinal cord compression). The typical recommendation initially was to continue bisphosphonates indefinitely at monthly intervals [8]. However, by 2003, osteonecrosis of the jaws or bisphosphonate-related osteonecrosis of the jaws (BRONJ) has been described as a new complication associated with their use [9]. Several studies have focused on the risk factors for developing BRONJ. Treatment with high potency (nitrogenated) intravenous bisphosphonates such as zoledronic acid and pamidronate, and dental extractions are important risk factors. The duration of bisphosphonate treatment, the number of infusions, and the total infusion hours may also be risk factors for BRONJ [10, 11].

We report a case of MM adjacent to a mandibular dental implant in a patient who developed BRONJ in the same area after intravenous zoledronate treatment. To our knowledge, this is the first case reported with this particular and interesting evolution.

## 2. Case Report

**Figure 1:**
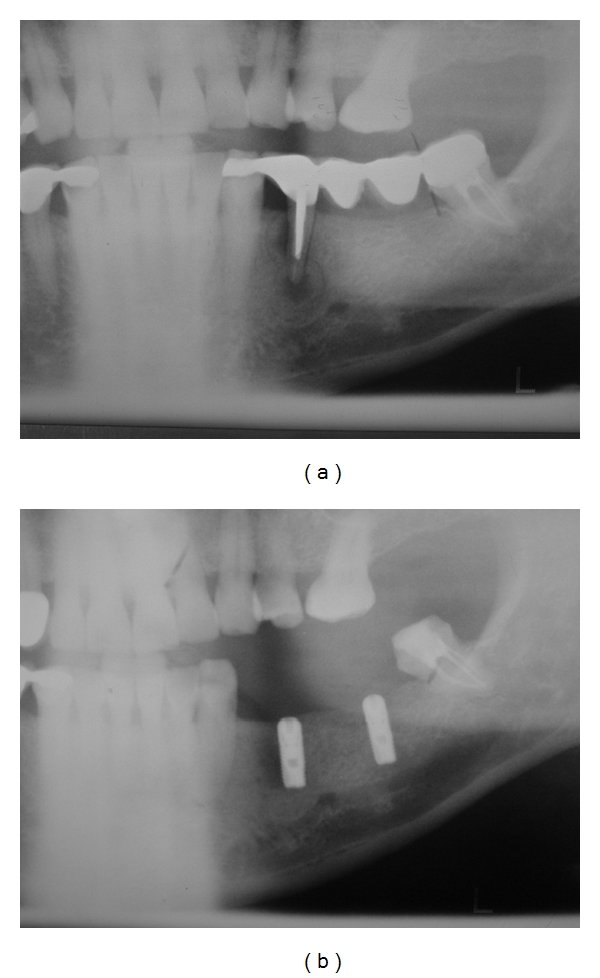
(a) Panoramic radiograph revealing periapical radiolucency in endodontically treated left mandibular premolar, when patient was completely healthy (March 2006). (b) Panoramic radiograph showing dental implants before final restoration. Implants were completely osseointegrated, and no mandibular lesions were observed (September 2006).

A 59-year-old Caucasian male patient was referred to our department in March 2007 for mandibular evaluation. In April 2006, when the patient was completely healthy, he underwent extraction of first left mandibular premolar with failed endodontic treatment (Figure 1(a)) and two intraosseous implants placement in the left mandibular molar region one month after. Dental implants were placed using a two-step process, and, six months after (October 2006), were uncovered and restored, uneventfully (Figure 1(b)).

**Figure 2:**
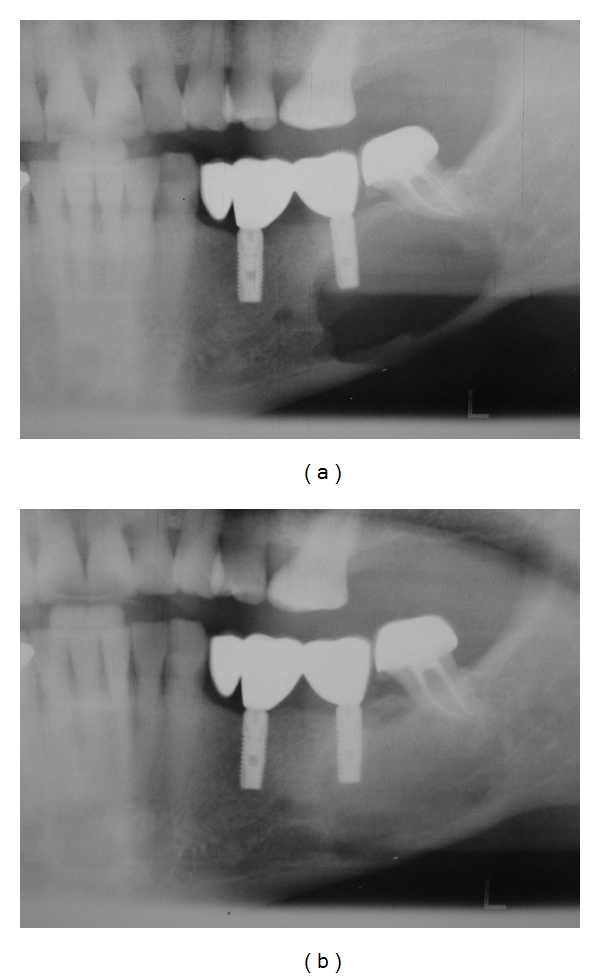
(a) Panoramic radiograph showed a large lytic lesion adjacent to the left implant and second left mandibular molar, diagnosed as MM (March 2007). (b) A new panoramic radiograph performed seven months after starting therapy revealed a complete regression of the mandibular lesion, supporting previous diagnosis of mandibular involvement of MM.

The patient received the diagnosis of MM in February 2007 (IgA lambda, Durie and Salmon's stage III A, ISS 3). When MM was diagnosed, the patient showed widely skeletal affection, including cranial calotte, thoracic and lumbar vertebral spine. Calcium, creatinine, and hemoglobin were in normal levels. The hematologist referred the patient to our department in March 2007 because of mandibular pain and swelling. Clinical examination revealed left mandibular cortical expansion without bone exposure or fistula in the affected area. No labial paresthesias were referred. A panoramic radiograph showed a large radiolucency adjacent to the left implant and second left mandibular molar (Figure 1(a)). A diagnosis of mandibular involvement of MM was proposed. As induction therapy, the treatment included six alternating cycles of vincristine, carmustine, melphalan, cyclophosphamide, prednisone (VBMCP) and vincristine, carmustine, doxorubicin and dexamethasone (VBAD) (each five/four weeks, resp.), ending in July 2007. The patient developed a very good partial response, without plasmatic cells in bone marrow and monoclonal pick in electrophoresis, but with positive immunofixation in serum. Consolidation therapy consisted in melphalan, alpha interpheron 2b, and autologous stem cell transplantation, carried out in October 2007. The patient also received 17 doses of Zoledronic Acid (4 mg/dose, once per month). A new panoramic radiograph performed seven months after starting therapy revealed a complete regression of the mandibular lesion (Figure 2), supporting previous diagnosis of mandibular involvement of MM. 

**Figure 3:**
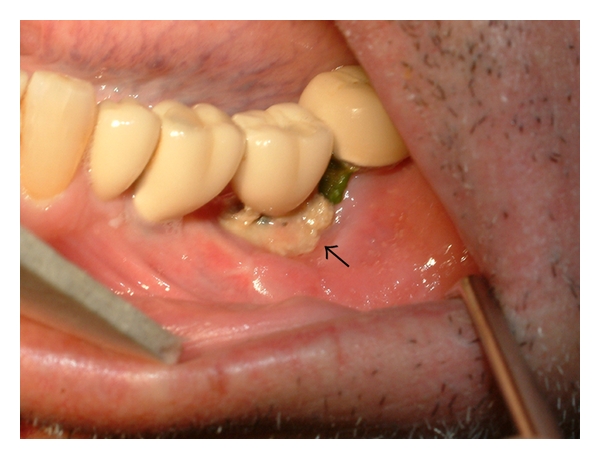
An extensive area of necrotic bone (arrow) is exposed adjacent to the left implant with purulent discharge (September 2008).

**Figure 4:**
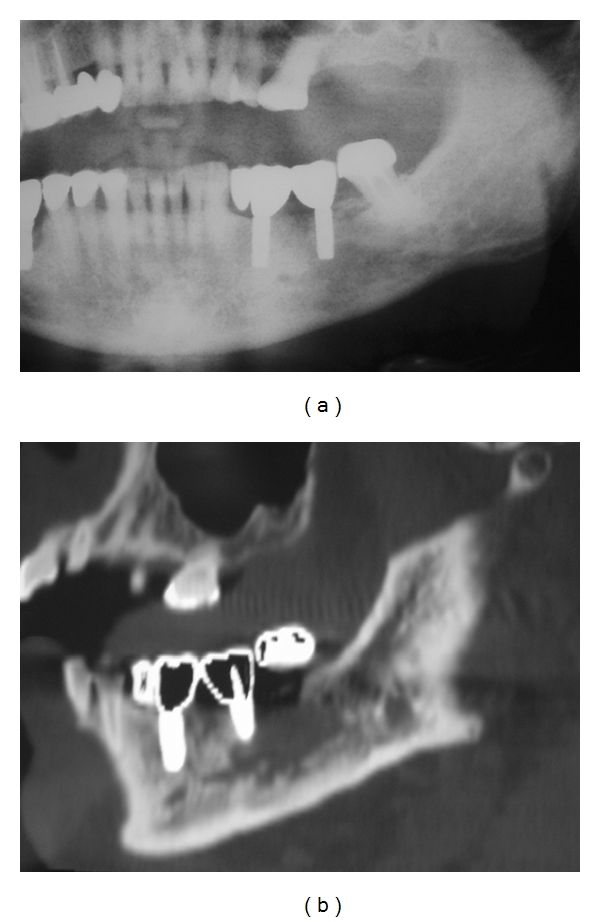
(a) A panoramic radiography showing only low-diffusion radiolucency around the left implant. (b) Computerized tomography image revealing necrotic bone involving left mandibular implant (September 2008).

**Figure 5:**
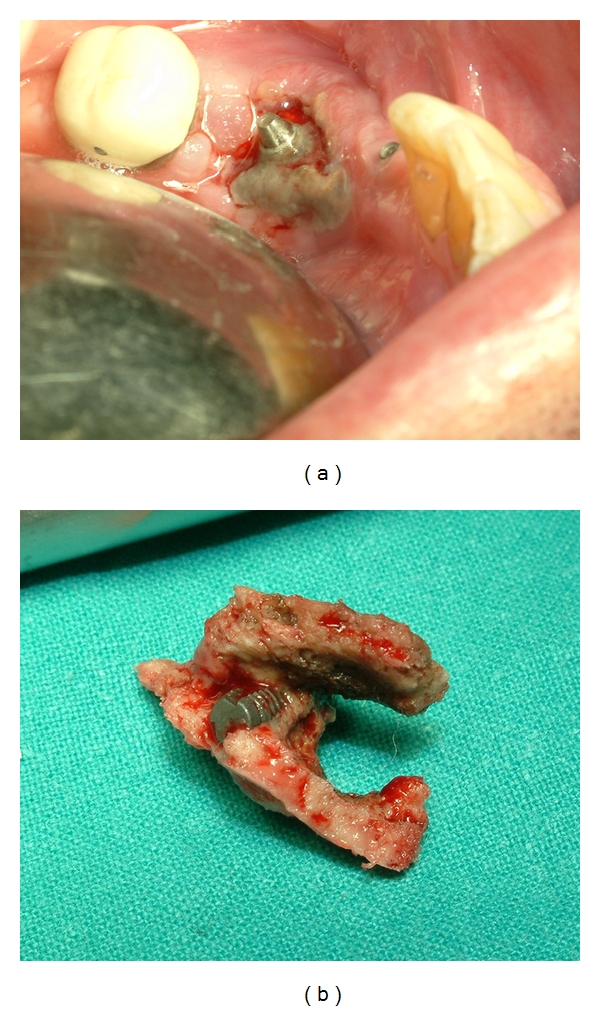
(a) Exposed necrotic bone around the left implant after three months of antibiotherapy. (b) Image of the resected specimen of bone sequestrum including left implant.

The patient presented again in September 2008 complaining of discomfort in the left mandibular molar area. Left inferior labial paresthesia was referred. Intraoral examination revealed that the alveolar bone adjacent to the left implant was exposed with a purulent discharge (Figure 3). The panoramic radiography provided poor diagnostic information, showing only low-diffusion radiolucency in the affected area (Figure 4(a)). Computerized tomography revealed low- and high-density areas in the alveolar process involving left mandibular implant and giving an aspect of necrotic bone (Figure 4(b)). Treatment consisted of simultaneous amoxicillin (4 gr/day) and clavulanate (250 mg/day) with chlorhexidine mouthwash. A biopsy of the lesion performed shortly thereafter showed bone necrosis with superficial contamination with oral organisms. Electrophoresis was absolutely normal. No evidence of MM was detected. A diagnosis of BRONJ was made. After 3 months, the pain did not disappear and bone exposure had not reduced (Figure 5(a)). Then, the mobile fragment of exposed necrotic bone including left implant was removed under local anesthesia (Figure 5(b)). The specimen was submitted for microscopic evaluation, and diagnosis was bone sequestrum with microbial colonies of *Actinomyces*. 

After 1 month, the pain had almost completely disappeared. The lesion was asymptomatic and soft tissues healed completely. At present, the patient is still under antineoplastic therapy (bortezomib and dexamethasone) for refractary vertebral MM, but zoledronate therapy has been discontinued.

## 3. Discussion

Current dental and orthopedic titanium implants have been developed based on the concept “bone-titanium integration” and are so-called “osseointegrated implants”. In addition to the bone interface, however, a considerable area of the implant surface may initially be in intimate contact with bone marrow. Possible interactions between implant surfaces and bone marrow cells could be a critical question in patients affected by hematologic malignancies. Rahal et al. [12] presented an experimental model placing titanium implants in the femur and bone marrow of mice. After 5 months after implantation, the titanium surface remained in direct contact with bone marrow cells. Prominent giant multinucleated cells with phagocytic inclusion, not seen in normal bone marrow, lined the interface. Then, reactions to foreign bodies within the bone marrow itself could affect the immune capacity of the host or predispose to potentially neoplastic dysregulation of bone marrow cells. 

Otherwise, a more recent experimental work in mice [13] provides no evidence to suggest that titanium implants in contact with mouse bone marrow carry a long-term risk of focal hemopoietic perturbations or marrow-derived B lineage neoplasm. The authors described a harmonious “myelointegration”. However, they found that an implant-marrow interface with associated giant cells persists for at least 1.5 years. Precursor B cells show early increases in number and proliferative activity. At later intervals, however, they do not differ significantly from controls, and there are no perturbations in spatial localization of either B lineage cells or DNA-synthesizing hemopoietic cells. Thus, myelointegration of dental implants in a previous healthy patient who develops a MM during osseointegration is a difficult and interesting question. There is a single case reported in the literature of a patient affected by a plasmacytoma of the spine and developed a new plasmacytoma of the mandible 3 years subsequent to the insertion of a dental implant. This second solitary lesion occurred 15 years after the first one, and without signs of conversion to MM [14]. 

As the jaws have a faster bone turnover rate (10 times higher than other bones), this requires adaptability and upregulation of the osteoblast and osteoclast function [15]. Otherwise, previous cases of plasma cell disorders preceded by skeletal trauma have been described [16–18]. Hussein et al. [18] hypothesized that trauma to the skeletal system could cause the release of cytokines leading to the proliferation of plasma and stromal cells in the bone. Thus, dental implants could have been represented a mandibular trauma and produced continued inflammation leading to overstimulation of plasma cells in the case reported. 

A single case of BRONJ around dental implants related to bone metastases of breast cancer has been reported [19], but the development of BRONJ adjacent to a dental implant in the same area previously affected by MM represents an extremely rare condition not previously published. Unfortunately, a single case report has made it difficult to determine cause and be confident of its behavior.
